# Selective inhibition of TNF-*α*-induced activation of mitogen-activated protein kinases and metastatic activities by gefitinib

**DOI:** 10.1038/sj.bjc.6602548

**Published:** 2005-04-19

**Authors:** Y Ueno, H Sakurai, M Matsuo, M K Choo, K Koizumi, I Saiki

**Affiliations:** 1 Division of Pathogenic Biochemistry, Institute of Natural Medicine, Toyama Medical and Pharmaceutical University, 2630 Sugitani, Toyama 930-0194, Japan; 2 The 21st century COE Program, Toyama Medical and Pharmaceutical University, Toyama 930-0194, Japan; 3 Department of Anatomy, Faculty of Medicine, Toyama Medical and Pharmaceutical University, Toyama 930-0194, Japan

**Keywords:** gefitinib, tumour necrosis factor alpha, epidermal growth factor, hepatocellular carcinoma, signalling pathways, metastases

## Abstract

We have reported that the selective epidermal growth factor receptor (EGFR) tyrosine kinase inhibitor, gefitinib (‘Iressa’, ZD1839), suppressed intrahepatic metastasis of hepatocellular carcinoma CBO140C12 cells. In this study, we focused on the tumour necrosis factor-*α* (TNF-*α*) signalling pathways. Real-time reverse transcription–polymerase chain reaction showed that TNF-*α* mRNA was expressed in large quantities in the implanted tumour. Gefitinib inhibited EGF- but not hepatocyte growth factor (HGF)-induced activation of mitogen-activated protein kinase (MAPK) cascades, suggesting selectivity of the inhibitor. However, gefitinib inhibited the TNF-*α*-induced activation of MAPKs and Akt. In addition, TNF-*α*-induced metastatic properties including adhesion to fibronectin, mRNA expression of integrin *α*v, production of matrix metalloproteinase-9 and invasion were inhibited by gefitinib without affecting cell proliferation. Furthermore, the TNF-*α*-induced responses except for NF-*κ*B activation were blocked by metalloprotease inhibitors, suggesting that gefitinib inhibited the transactivation of EGFR induced by TNF-*α*. These results suggest that the TNF-*α* signalling pathway is a possible target of gefitinib in suppressing the intrahepatic metastasis of hepatocellular carcinoma.

High expression of epidermal growth factor receptor (EGFR) has been observed in a variety of tumours, including the lung and liver (Ito *et al*, 2001; Nicholson *et al*, 2001), which has been shown to correlate with disease progression, poor survival, poor response to therapy and the development of resistance to cytotoxic agents (Arteaga, 2002; Ritter and Arteaga, 2003). Upon ligand binding, EGFR dimerisation triggers protein kinase activity of the intracellular tyrosine kinase domain (Baselga, 2002). Gefitinib (‘Iressa’, ZD1839 (‘Iressa’ is a trademark of the AstraZeneca group of companies)), an EGFR tyrosine kinase inhibitor, exhibits a broad antitumour spectrum against human cancers *in vitro* and demonstrated therapeutic benefit in patients with non-small-cell lung cancer (Ciardiello *et al*, 2000; Sirotnak *et al*, 2000; Herbst and Bunn, 2003). There is no correlation between the antitumour activity of gefitinib and EGFR expression level; however, recent research revealed that gefitinib-responsive lung cancers harbour somatic mutations within the EGFR kinase domain (Lynch *et al*, 2004; Paez *et al*, 2004; Sordella *et al*, 2004).

Tumour necrosis factor-*α* (TNF-*α*) was first identified as the macrophage-derived product responsible for tumouricidal activity (Matthews, 1978; Matthews and Watkins, 1978; Pennica *et al*, 1985). However, extensive research during past years has made it apparent that TNF-*α* enhances the incidence of metastasis in several tumour models (Orosz *et al*, 1993; Qin *et al*, 1993; Kitakata *et al*, 2002; Mochizuki *et al*, 2004; Tomita *et al*, 2004). The role of endogenous TNF-*α* in the metastatic process remains to be clarified; however, TNF-*α* has been found to increase the expression of adhesion molecules and invasive molecules, including intracellular adhesion molecule-1 (ICAM-1) and matrix metalloproteinase-9 (MMP-9) (Aggarwal *et al*, 2002). In addition, we have recently reported that stimulation of cultured colon 26 cells with TNF-*α* promotes lung metastasis through the extracellular signal-regulated kinase (ERK) signalling pathway (Choo *et al*, 2005).

Epidermal growth factor receptor transactivation by ligand-independent intracellular signalling mechanisms and metalloprotease-mediated processing of the EGF-like ligands has been investigated in the last couple of years (Fischer *et al*, 2003). Recently, the transactivation of EGFR by TNF-*α*-induced metalloprotease processing of TGF-*α* has been demonstrated in hepatocytes and mammary epithelial cells (Argast *et al*, 2004; Chen *et al*, 2004). These findings raise a possibility that TNF-*α* signalling pathways are potential targets for the antitumour activity of gefitinib.

Recently, we have shown that gefitinib is effective in inhibiting intrahepatic metastasis of murine hepatocellular carcinoma CBO140C12 cells by blocking EGFR-dependent metastatic properties (Matsuo *et al*, 2003). In this study, we found that gefitinib also inhibited the TNF-*α*-induced activation of mitogen-activated protein kinase (MAPK) cascades and Akt as well as TNF-*α*-induced metastatic properties *in vitro* possibly by inhibiting EGFR transactivation.

## MATERIALS AND METHODS

### Reagents

Gefitinib was kindly provided by AstraZeneca (Macclesfield, UK). It was dissolved in DMSO for the *in vitro* study. Recombinant murine EGF were purchased from Upstate Biotechnology and murine hepatocyte growth factor (HGF) and human TNF-*α* were purchased from Genzyme/Techne. Metalloprotease inhibitors, GM6001, GM6001 negative and TAPI-1, were purchased from Calbiochem, Darmstadt, Germany.

### Intrahepatic metastasis model by orthotopic implantation

Female 5-week-old specific pathogen-free B6C3F1 mice were purchased from Japan SLC (Hamamatsu, Japan). The mice were maintained under specific pathogen-free conditions and used according to institutional guidelines. Orthotopic implantation of CBO140C12 tumour fragments into mouse liver was performed as described previously (Sawada *et al*, 2001; Matsuo *et al*, 2003). The mice were killed on day 14 and total RNA was prepared from the liver and primary tumour mass, and then subjected to real-time reverse transcription–polymerase chain reaction (RT–PCR) as described below.

### Reverse transcription–polymerase chain reaction

Total RNA from CBO140C12 cells was extracted using the RNeasy Mini Kit (QIAGEN, Hilden, Germany). First strand cDNA was prepared from the total RNA (1 *μ*g) using oligo(dT) primer and SuperScript II reverse transcriptase (Invitrogen, Carlsbad, USA). The cDNA was conducted in a 25 *μ*l final volume mixture containing primer, probe and TaqMan Universal PCR Master Mix. Probes were labelled with fluorescent reporter dye at the 5′-end (glyceraldehydes-3-phosphate dehydrogenase (GAPDH): VIC; TNF-*α*: FAM; MMP-9: FAM) and a quencher dye at the 3′-end (GAPDH: TAMRA; TNF-*α*: MGB; MMP-9: MGB). Plates were analysed on a TaqMan ABI Prism 7700 Sequence Detector (Applied Biosystems, Carlsbad, USA). Cycling parameters were: 50°C for 2 min, 95°C for 10 min, 40 cycles for GAPDH and TNF-*α*, 50 cycles for MMP-9 at 95°C for 15 s and 60°C for 1 min. Cycle threshold detection was converted into number of cDNA contents in the starting material and a standard curve was constructed using the known amounts of cDNA. Test gene mRNA values were extrapolated from the standard curve and expressed in arbitrary units.

Amplification of integrin *α*v subunit mRNA was performed by standard RT–PCR using specific oligonucleotide primers and an EX *Taq* PCR kit (Takara-bio Co., Ltd., Shiga, Japan). The sequences of the primers were as follows: integrin *α*v, 5′-CAAGCTCACTCCCATCAC-3′ and 5′-GGGTGTCTTGATTCTCAAAGGG-3′; GAPDH, 5′-GGTGAAGGTCGGTGTGAACGGATTT-3′ and 5′-GATGCCAAA GTTGTCATGGATGACC-3′. Polymerase chain reaction was preformed in a thermocycler for specified cycles of denaturation (94°C, 30 s), annealing (60°C, 60 s) and extension (72°C, 90 s). The PCR products were electrophoresed on 1.2% agarose gels and detected by ethidium bromide staining.

### Cell culture

The CBO140C12 murine hepatocellular carcinoma cell line was kindly provided by Dr K Ogawa (Asahikawa Medical College, Japan) and maintained in DMEM : F-12 supplemented with 10% FCS, 320 mg l^−1^ L-glutamine. A549 cells were maintained in DMEM supplemented with 10% FCS.

### Cell proliferation assay

Cells (1 × 10^4^ cells well^−1^) were seeded in 100 *μ*l of medium containing 0.5%. FCS in 96-well plates and allowed to adhere for 24 h. After preincubation with 90 *μ*l of medium containing gefitinib (final concentration 1 *μ*M) for 15 min, cells were stimulated with 10 *μ*l of medium containing TNF-*α* (final concentration 10 ng ml^−1^) for 12 or 72 h. Cell proliferation was determined by using a cell counting kit (Dojindo).

### Western blot analysis

Cells were cultured in a medium containing 0.5% FBS for 24 h. After indicated treatment, cell lysates were prepared with sample buffer (25 mM Tris-HCl (pH 6.8), 5% w v^−1^ glycerol, 1% w v^−1^ SDS, 0.05% w v^−1^ bromophenol blue) and were subjected to sodium dodecyl sulphate–polyacrylamide gel electrophoresis (SDS–PAGE) and transferred to Immobilon-P membranes (Millipore). Blots were probed using primary antibodies described above and horseradish peroxidase-conjugated secondary antibodies (DAKO, Glostrup, Denmark) followed by enhanced chemiluminescence (Amersham, Piscatway, USA). Antibodies against EGFR and phospho-EGFR, phospho-ERK, phospho-c-Jun-N-terminal kinase (JNK), phospho-Akt, phospho-p38 and phospho-p65 were purchased from Cell Signaling Technology, Beverly, USA and anti-p38, JNK, p65 and Akt antibodies were obtained from Santa Cruz Biotechnology, California, USA.

### Adhesion assay

Cells in 0.1% BSA medium were pretreated with gefitinib for 15 min and then stimulated with TNF-*α* for 12 h. In all, 2 × 10^4^ cells were seeded on to the 96-well plate precoated with 1 *μ*g of fibronectin. After incubation for 25 min, attached cells were stained with 0.5% crystal violet. The cells were lysed with 30% acetic acid, and the absorbance was measured at 590 nm.

### Invasion assay

The invasion assay was performed using Transwell culture chambers (Corning Costar). Polyvinylpyrrolidone-free polycarbonate filters with an 8.0 *μ*m pore size (Neuclepore) were precoated with 1 *μ*g of fibronectin on the lower surface, and then 5 *μ*g of Matrigel was applied to the upper surface of the filter. Cells in 0.1% BSA medium were pretreated with gefitinib for 15 min, and then stimulated with TNF-*α* for 12 h. In all, 3 × 10^4^ cells were added to the upper compartment of the chamber and incubated for 6 h at 37°C. The cells were stained with haematoxylin and eosin and were counted using the mean of five windows (× 400 magnification) per filter.

### Gelatin zymography

Gelatin zymography was performed as previously described (Matsuo *et al*, 2003) with some modifications. Briefly, the conditioned media was concentrated using Centricon (Millipore) according to the manufacturer's instructions and applied to 7.5% SDS–polyacrylamide gels copolymerised with gelatine (0.1% w v^−1^) and incubated at 37°C for 24 h. Enzyme-digested regions were quantified by the Chemi Doc XRS system (Bio-Rad).

### Statistical analysis

The significance of differences between groups was determined by applying Student's two-tailed *t*-test.

## RESULTS

### Enhanced expression of TNF-*α* mRNA in tumour-implanted liver

**Figure 1:**
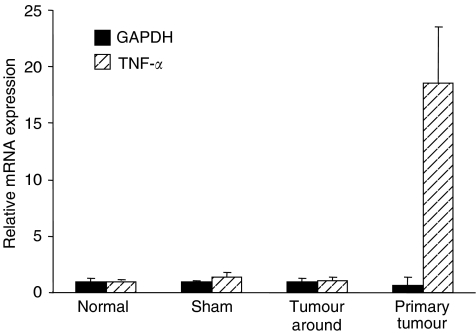
mRNA expression of TNF-*α* in the liver and tumour tissues from the B6C3F1 mouse. B6C3F1 mice were given implantation with a tumour fragment of CB140C12 cells, sham operation. Normal mice were given no operation. Total RNAs were prepared from primary tumors, liver tissues around the tumour, the sites of sham operation and normal livers, and real-time RT–PCR was performed for quantification of relative mRNA expression of TNF-*α* and GAPDH. All data are represented as mean±s.d. of three mice.

We have previously reported that gefitinib inhibits the spontaneous intrahepatic metastasis of hepatocellular carcinoma by blocking the EGFR-mediated metastatic properties (Matsuo *et al*, 2003). Here, we focused on the TNF-*α* signalling pathway. It has been demonstrated that inflammatory cytokines including TNF-*α* play critical roles in tumour metastasis. Therefore, we first tried to detect mRNA expression of TNF-*α* in the intrahepatic metastasis model using real-time RT–PCR (Figure 1). High-level expression could be detected in the primary tumour mass. In contrast, mRNA expression of TNF-*α* in the liver around the tumour was comparable with normal and sham-operating liver. These results confirm tumour-induced inflammatory reactions in the implanted primary tumour.

### Effects of gefitinib on EGF-, HGF- and TNF-*α*-induced signalling pathways

**Figure 2:**
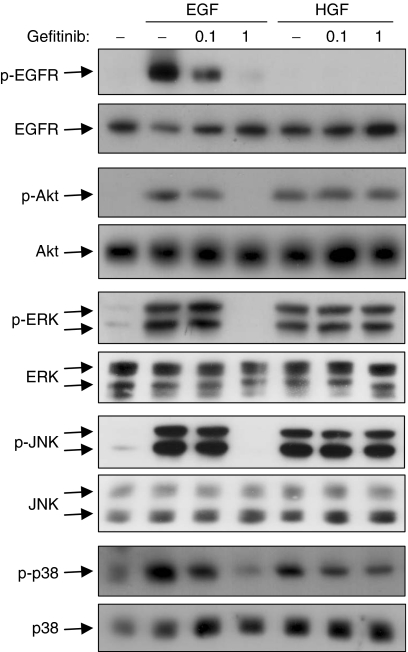
Selective inhibition of EGF-induced signalling pathways by gefitinib in CBO140C12 cells. Serum-starved CBO140C12 cells were pretreated for 15 min with the indicated concentrations (*μ*M) of gefitinib, followed by the stimulation with 10 ng ml^−1^ EGF, or 10 ng ml^−1^ HGF for 5 min. Phospho-EGFR, phospho-Akt, phospho-ERK, phospho-JNK and phospho-p38 were determined by Western blotting using phospho-EGFR (Tyr1068), phospho-Akt (Ser473), phospho-ERK (Thr202, Tyr204), phospho-JNK (Thr183, Tyr185) and phospho-p38 (Thr180, Tyr182) antibodies, respectively.

Gefitinib is known as a selective EGFR tyrosine kinase inhibitor. In fact, gefitinib inhibited EGF-induced EGFR autophosphorylation (Tyr-1068) as well as the downstream signalling pathways including Akt, ERK, JNK and p38 MAPK in CBO140C12 cells (Figure 2). In contrast, HGF-induced activation of these kinases was not affected by gefitinib (Figure 2). The selectivity of the signalling pathways was correlated with our previous results, which showed that gefitinib inhibited EGF- but not HGF-induced chemotactic migration of CBO140C12 cells (Matsuo *et al*, 2003).

**Figure 3:**
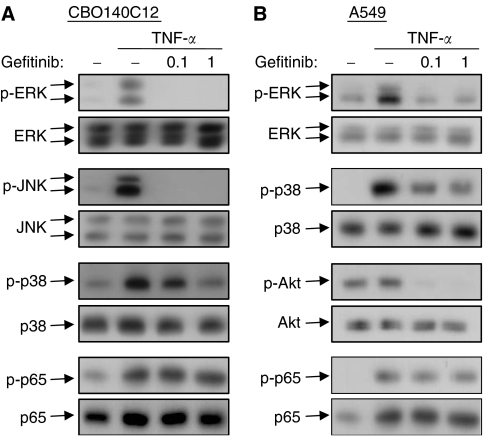
Inhibition of TNF-*α*-induced MAPK activation by gefitinib in (**A**) CBO140C12 and (**B**) A549 cells. Cells were treated with the indicated concentrations (*μ*M) of gefitinib for 15 min, followed by the stimulation with 10 ng ml^−1^ TNF-*α* for 10 min. Phospho-ERK, phospho-JNK, phospho-p38, phospho-Akt and phosphor-NF-*κ*B p65 (Ser-536) were determined by Western blotting.

The signalling molecules tested above are also involved in the TNF-*α* signalling, therefore, we next examined the effects of gefitinib on the TNF-*α*-induced responses. Interestingly, TNF-*α*-induced activation of MAPKs was significantly inhibited by gefitinib in CBO140C12 cells (Figure 3A). Phosphorylation of Akt was not affected by TNF-*α* and gefitinib (data not shown). It should be noted that the inhibition was more potent compared with the inhibitory activity against EGF-induced MAPK activation. Gefitinib completely inhibited TNF-*α*-induced activation of ERK and JNK at 0.1 *μ*M (Figure 3A); however, only a slight inhibition was observed in EGF-induced MAPK activation at the same concentration (Figure 2). In addition, activation of MAPK and Akt was impaired in the presence of gefitinib in human cancer cell lines, A549 (Figure 3B). In contrast, TNF-*α*-induced phosphorylation of NF-*κ*B p65 subunit at Ser-536, an essential event for activation of NF-*κ*B (Sakurai *et al*, 1999), was not affected by gefitinib in these cell lines (Figure 3A and B), indicating that gefitinib did not block the TNF-*α* responses at the receptor level.

### Effects of gefitinib on TNF-*α*-induced metastatic properties

**Figure 4:**
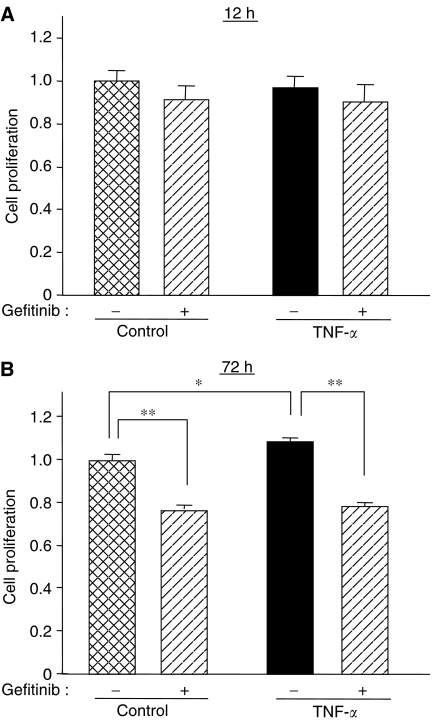
Effects of gefitinib and TNF-*α* on cell proliferation. CBO140C12 cells were incubated with 1 *μ*M gefitinib and/or TNF-*α* for 12 h (**A**) or 72 h (**B**), and cell proliferation was determined by WST-1 assay. ^*^P<0.05, ^**^P<0.01.

It has been reported that TNF-*α* induces hepatocyte proliferation (Argast *et al*, 2004). We examined the effect of TNF-*α* on the growth of CBO140C12 cells. Stimulation with TNF-*α* for 72 h slightly increased cell proliferation (Figure 4B). We have previously reported that gefitinib inhibits cell proliferation along with caspase-3 activation in CBO140C12 cells (Matsuo *et al*, 2003). The apoptotic activity of gefitinib was also detected even in the presence of TNF-*α* (Figure 4B). However, neither gefitinib nor TNF-*α* affected the growth during a 12-h incubation period (Figure 4A).

**Figure 5:**
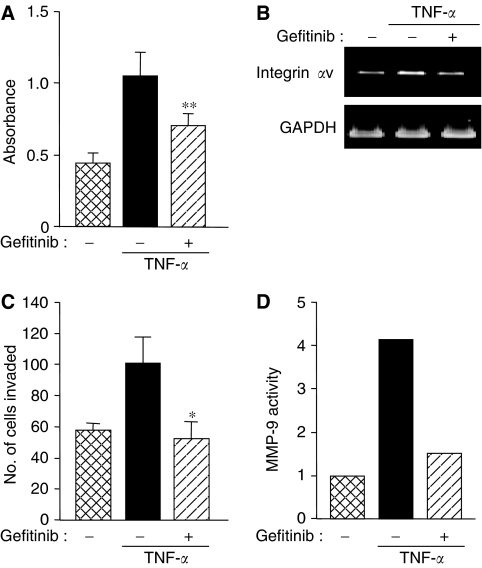
Effects of gefitinib on TNF-*α*-induced metastatic properties. Cells were pretreated with 1 *μ*M gefitinib for 15 min, followed by stimulation with 10 ng ml^−1^ TNF-*α*. (**A**) After a 12-h incubation, cells were harvested and incubated in the wells precoated with fibronectin for 25 min. The adhesive cells were stained with crystal violet and absorbance was meseaured. (**B**) After incubation for 6 h, mRNAs encoding integrin *α*v and GAPDH were amplified by RT–PCR and visualized with ethidium bromide staining. (**C**) After a 12-h incubation, cells were harvested and seeded into the invasion chamber precoated with 1 *μ*g fibronectin on the lower surface and 5 *μ*g Matrigel on the upper surface of the filter. After a 6-h incubation, chambers were stained with haematoxylin and eosin and the numbers of cells invaded were counted under a × 400 microscope. (**D**) After a 12-h incubation, the conditioned medium was analysed using gelatin zymography. The activities were quantified by the Chemi-Doc XRS system. ^*^P<0.05, ^**^P<0.01.

Adhesion to the extracellular matrix and invasion across the matrix and basement membrane are the critical steps in tumour metastasis. CBO140C12 cells were stimulated with TNF-*α* for 12 h prior to the adhesion assay. TNF-*α* stimulated cell adhesion to fibronectin and the increased adhesion was blocked by gefitinib (Figure 5A). This is correlated with the inhibition of TNF-*α*-induced mRNA expression of integrin *α*v subunit, a counterpart of fibronectin, by gefitinib (Figure 5B). In addition, gefitinib inhibited TNF-*α*-induced mRNA expression (data not shown) and the gelatinase activity (Figure 5D) of MMP-9 as well as TNF-*α*-induced invasion of CBO140C12 cells (Figure 5C).

### Effects of metalloprotease inhibitors on TNF-*α*-induced cellular responces

**Figure 6:**
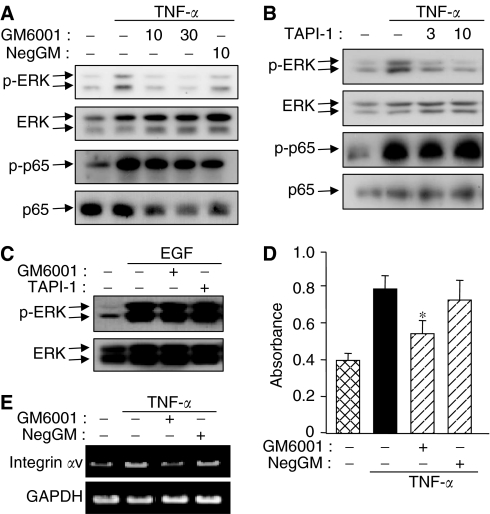
Effects of MMP inhibitors on TNF-*α*-induced MAPK activation and adhesion. Cells were pretreated with the indicated concentrations (*μ*M) of GM6001, GM6001 negative (NegGM) or TAPI-1 for 30 min, followed by stimulation with TNF-*α* (**A**, **B**) for 10 min, or EGF for 5 min (**C**). Phospho-ERK and phosphor-NF-*κ*B p65 were determined by Western blotting. (**D**) Cells treated with 10 *μ*M GM6001/NegGM and TNF-*α* for 12 h were tested in adhesion assay. (**E**) RT–PCR was performed using cells treated with 10 *μ*M GM6001/NegGM and TNF-*α* for 6 h. ^*^P<0.01.

To elucidate the possibility that responses to TNF-*α* in CBO140C12 cells are mediated by EGFR transactivaton, effects of metalloprotease inhibitors were examined. GM6001, a broad metalloprotease inhibitor, blocked TNF-*α*-induced phosphorylation of ERK1/2 (Figure 6A). Activation of JNK and p38 was also inhibited by GM6001 (data not shown), whereas phosphorylation of NF-*κ*B p65 at Ser-536 was not inhibited (Figure 6A). In contrast, NegGM, a structurally similar compound without metalloprotease inhibitory activity, did not inhibit TNF-*α*-induced phosphorylation of ERK1/2 as well as phosphorylation of NF-*κ*B (Figure 6A). It has recently been demonstrated that shedding of EGFR ligands is mediated by some members of the ADAMs family of metalloproteases, especially ADAM17 (Hinkle *et al*, 2004; Sahin *et al*, 2004). We therefore examined the effect of TAPI-1, an ADAMs inihibitor. TNF-*α*-induced phosphorylation of ERK1/2 was inhibited by TAPI-1 (Figure 6B). Both GM6001 and TAPI-1 did not inhibit EGF- and HGF-induced phosphorylation of ERK1/2 (Figure 6C and data not shown), indicating that these inhibitors selectively blocked the TNF-*α*-induced signalling pathways. Moreover, TNF-*α*-induced metastatic properties including the cell adhesion to fibronectin (Figure 6D) and mRNA expression of integrin *α*v (Figure 6E) were inhibited by GM6001. The selective inhibiton by metalloprotease inhibitors was similar to the inhibitory effects of gefitinib on the TNF-*α*-induced cellular responces.

## DISCUSSION

Epidermal growth factor receptor is a promising target for cancer therapy and a number of anti-EGFR agents have been developed (Blackledge and Averbuch, 2004). Preclinical investigations and clinical studies of gefitinib have shown the benefits of anti-EGFR therapy (Herbst and Bunn, 2003). It has recently been demonstrated that TNF-*α* induces EGFR transactivation via metalloproteinase-dependent release of EGFR ligands. Therefore, we have tired to examine the effects of gefitinib on the TNF-*α*-induced cellular responces.

There has been accumulating evidence that inflammatory mediators such as TNF-*α* promote malignant cell growth and metastatic potential (Kitakata *et al*, 2002; Lozano *et al*, 2003). Tumour metastasis is a complex process involving the release of tumour cells from a primary tumour, entering of the vascular or lymphatic circulation and extravasation to specific sites distant from original tumour. These processes require tumour cell attachment, migration, and invasion. In several tumour cell lines, TNF-*α* induced the expression of several different adhesive molecules including several integrin subunits, ICAM-1 and VCAM-1 (Orosz *et al*, 1993; Qin *et al*, 1993; Choo *et al*, 2005). Tumour necrosis factor-*α* also induced the activation of MAPKs, which has been shown to be involved in MMP-9 expression and invasion. In this study, we found that gefitinib inhibited not only EGF-induced but also TNF-*α*-induced activation of MAPKs and metastatic properties including adhesion and invasion. In addition, we have previously demonstrated that gefitinib inhibited intrahepatic metastasis of CBO140C12 cells (Matsuo *et al*, 2003). Abundant expression of TNF-*α* mRNA in primary tumour of orthotopically implanted CBO140C12 cells in the liver support the idea that TNF-*α*-meidated cellular activities are potential targets for the antimetastatic activity of gefitinib. Moreover, TNF-*α* is one of the angiogenic factors associated with tumour-induced neovascularisation (Wu *et al*, 2000) and gefitinib has been shown to inhibit EGFR-mediated migration and tube-like formation of human microvascular endothelial cells (Hirata *et al*, 2002). These results suggest that gefitinib affected TNF-*α* signalling pathways in both tumour and endothelial cells. In our previous observation, gefitinib inhibited the incidence of metastasis as well as the growth of primary tumour (Matsuo *et al*, 2003). Therefore, antiangiogenic activity by blocking the EGF- and TNF-*α*-induced reactions may be one way gefitinib exerts its antitumour activity.

Recently, TNF-*α* and EGF signalling pathways have been found to play a physiological function in TNF-*α* signalling (Hirota *et al*, 2001). It has been suggested that proteolytic release of transforming growth factor-*α*, one of the EGFR ligands, is one possible mechanism of EGFR transactivation by TNF-*α* (Argast *et al*, 2004; Chen *et al*, 2004). Argast *et al* proved that MMPs, especially ADAM17, is responsible for the transactivation of EGFR by TNF-*α*. Here we confirmed that an ADAMs inhibitor TAPI-1 inhibited the activation of TNF-*α*-induced MAPK activation. In addition, TNF-*α*-induced metastatic properties *in vitro* such as adhesion were also inhibited by a broad MMP inhibitor GM6001. These results demonstrated that TNF-*α*-induced metastatic properties were mediated via MMP activities. The fact that gefitinib inhibited the TNF-*α*-induced cellular responses suggested the TNF-*α*-induced EGFR transactivation by shedding EGFR ligands. Identification of the ligands and MMPs will provide more information for the mechanism of anti-TNF-*α* activity of gefitinib.

Tumour necrosis factor-*α* triggers several intracellular signalling pathways, in which MAPK cascades and NF-*κ*B are the main pathways. In contrast to MAPK cascades, phosphorylation of NF-*κ*B p65 was not inhibited by both gefitinib and MMP inhibitors. This is correlated with the finding that AG1478, an EGFR inhibitor, did not inhibit TNF-*α*-induced phosphorylation of I*κ*B*α*, another event essential for activation of NF-*κ*B, in hepatocytes (Argast *et al*, 2004). In addition, it has been reported that TNF-*α*-induced expression of chemokine *RANTES*, one of the NF-*κ*B target genes, was not blocked by the EGFR-neutralizing monoclonal antibody 225 in mammary epithelial cells (Chen *et al*, 2004). Collectively, our observations support the proposed model that TNF-*α*-induced NF-*κ*B activation is independent of EGFR (Argast *et al*, 2004; Chen *et al*, 2004).

In summary, we have demonstrated that gefitinib shows antimetastatic activity using an intrahepatic metastasis model, in which TNF-*α*-induced EGFR signalling are the possible targets. Recent genome-wide approaches identified genes that may be associated with sensitivity to gefitinib (Zembutsu *et al*, 2003; Lynch *et al*, 2004; Paez *et al*, 2004). The accumulating evidence of biochemical and genetic characterisation for the mechanism of action will provide more information for the effective clinical use of gefitinib.
